# Enhanced Catalytic Hydrogenation of Olefins in Sulfur-Rich Naphtha Using Molybdenum Carbide Supported on γ-Al_2_O_3_ Spheres under Steam Conditions: Simulating the Hot Separator Stream Process

**DOI:** 10.3390/ma17102278

**Published:** 2024-05-11

**Authors:** Hadj Abbas Abbas, Zahra Asgar Pour, Mohammed S. Alnafisah, Pablo Gonzalez Cortes, Mustapha El Hariri El Nokab, Ahmed Elshewy, Khaled O. Sebakhy

**Affiliations:** 1 Laboratoire de géologie du Sahara, Université Kasdi Merbah Ouargla, Ouargla 30000, Algeria; abbas.ha@univ-ouargla.dz; 2 Research and Development Department, Kisuma Chemicals, Billitonweg 7, 9641 KZ Veendam, The Netherlands; 3 King Abdulaziz City for Science and Technology (KACST), Riyadh 12354, Saudi Arabia; manafisah@kacst.gov.sa; 4 Engineering and Technology Institute Groningen (ENTEG), University of Groningen, Nijenborgh 4, 9747 AG Groningen, The Netherlands; p.andresgonzalezcortes@gmail.com; 5 Laboratorio de Nanocelulosa y Biomateriales, Departamento de Ingeniería Química, Biotecnología y Materiales, Facultad de Ciencias Físicas y Matemáticas, Universidad de Chile, Avenida Beauchef 851, Santiago 8330111, Chile; 6 Department of Chemistry, Michigan State University, East Lansing, MI 48824, USA; elhariri@msu.edu; 7 Department of Pharmaceutical Organic Chemistry, Faculty of Pharmacy, Cairo University, Cairo 11562, Egypt; ahmed.elshewy@pharma.cu.edu.eg; 8 Laboratory for Chemical Technology (LCT), Department of Materials, Textiles and Chemical Engineering, Ghent University, Technologiepark 125, 9052 Ghent, Belgium; 9 Department of Chemical and Petroleum Engineering, University of Calgary, Calgary, AB T2N 1N4, Canada

**Keywords:** molybdenum carbide, olefin selective hydrogenation, hot separator, naphtha, sulfur, steam tolerance

## Abstract

Spheres comprising 10 wt.% Mo_2_C/γ-Al_2_O_3_, synthesized through the sucrose route, exhibited unprecedented catalytic activity for olefin hydrogenation within an industrial naphtha feedstock that contained 23 wt.% olefins, as determined by supercritical fluid chromatography (SFC). The catalyst demonstrated resilience to sulfur, exhibiting no discernible deactivation signs over a tested 96 h operational period. The resultant hydrogenated naphtha from the catalytic process contained only 2.5 wt.% olefins when the reaction was conducted at 280 °C and 3.44 × 10^6^ Pa H_2_, subsequently blended with Athabasca bitumen to meet pipeline specifications for oil transportation. Additionally, the carbide catalyst spheres effectively hydrogenated olefins under steam conditions without experiencing any notable hydrogenation in the aromatics. We propose the supported carbide catalyst as a viable alternative to noble metals, serving as a selective agent for olefin elimination from light petroleum distillates in the presence of steam and sulfur, mitigating the formation of gums and deposits during the transportation of diluted bitumen (dilbit) through pipelines.

## 1. Introduction

There exists a persistent demand to produce light petroleum distillates, specifically naphthas and gasolines, derived from unconventional feedstocks [1,2]. Various upgrading technologies, including thermal and catalytic cracking processes, are utilized due to their low investment costs [3,4,5,6,7,8,9,10]. Despite their advantages, both methods yield naphthas with high levels of deleterious olefinic compounds [11,12]. These olefins, present in the distillates, adversely impact fuel storage stability and physico-chemical characteristics, undergoing oxidation/polymerization upon exposure to air [11]. This results in the formation of high molecular weight deposits and acidic components, causing pipeline blockages and fouling in heat exchangers and other refinery equipment [13,14,15]. Moreover, during the hydro-treatment process in a refinery, naphtha and gasoline undergo sulfur removal at elevated temperatures, potentially reaching up to 500 °C [16]. At this temperature, olefins undergo polymerization, leading to coke formation, catalyst deactivation, and temperature runaway in the reactor [17]. The Canadian Association of Petroleum Producers (CAPP) notes that pipeline companies have currently established stringent regulations for accepting synthetic oil derived from Alberta Bitumen.

Hence, there is a significant imperative to selectively hydrogenate the reactive olefinic species present in naphthas at an earlier stage through a cost-effective catalytic process. This aims to eliminate olefins, facilitating the blending of hydrogenated naphtha with bitumen to mitigate/reduce viscosity for pumping and adhere to pipeline specifications for oil transportation. The target is to achieve a final olefinic content of less than 1 wt.% in the diluted bitumen (dilbit) [7,8]. Cracked naphtha, derived from FCC/Coker units, stands out as a prominent contributor to pressure drops in the industry [13,14,15]. Naphthas generated in refineries harbor highly reactive olefinic molecules, necessitating precautions against prolonged storage or exposure to air. These molecules exhibit a proclivity to generate resinous, polymeric, and non-volatile deposits, thereby altering the final stability and physico-chemical properties of the naphtha [13,14]. Additionally, these olefinic materials can induce fouling in the heat exchanger tubes, resulting in the formation of coke deposits and causing pressure drop issues. If olefinic compounds enter the reactor without undergoing prior reactions in the heat exchanger, they pose significant operational stability risks. These olefins tend to react with each other or with absorbed atmospheric oxygen, forming extended chain polymers referred to as “gums” [13,14,15]. Accumulation and agglomeration of these gums on the reactor bed pores lead to the formation of rigid coke layers, rapidly fouling the reactor and causing excessive pressure drops, which is a key operational problem in the industry.

Carbides, including those of Mo, W, Nb, or Ta, represent a substantial and captivating category of materials within the realm of catalysis, garnering research interest over numerous years [18,19,20,21,22,23,24,25,26,27,28,29,30,31]. In their 1973 publication, Levy and Boudart [18] demonstrated the platinum-like behavior of tungsten carbides in certain reactions, such as hydrogenation, marking the initiation of a new phase of investigation into these materials. These interesting materials have shown high catalytic activity in hydrogenation reactions [19,21,22]. Molybdenum carbides, whether in bulk or supported form, with or without promotion, have undergone testing and exhibited activity in the hydrogenation of aromatics [22,32] and olefins [32]. Furthermore, they have demonstrated excellent sulfur tolerance during thiophene hydrodesulfurization [20,25,27] and steam tolerance [33], inevitably providing conclusive evidence of water dissociation and hydrogenation on molybdenum carbide nanocatalysts for hydroprocessing reactions.

**Scheme 1:**
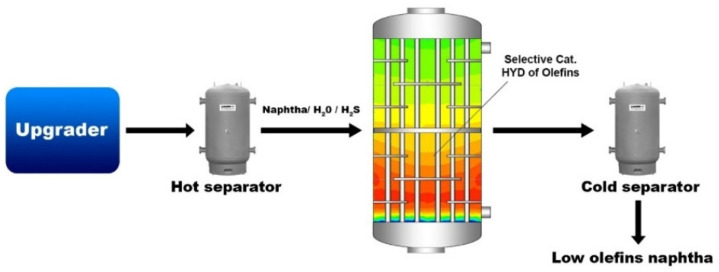
Representation of olefin elimination in naphtha streams containing water and sulfur. A process simulating the hot separator stream.

The primary objective of the present study was to achieve the selective hydrogenation of olefins present in an industrial naphtha produced from the catalytic steam cracking (CSC) process by employing our recently developed and cost-effective molybdenum carbide catalyst [32] at moderate temperatures and pressures, with minimal aromatics hydrogenation to keep a high-octane value. The resultant hydrogenated naphtha holds the potential for utilization as fuel or as a diluent in bitumen to reduce viscosity, aligning with pipeline specifications for the transportation of dilbit. An additional objective involved the selective hydrogenation of olefins in naphtha streams containing water and sulfur, such as those obtained from the hot separator in a CSC process, as depicted in Scheme 1. This catalytic process could be commercialized for the specific hydrogenation of olefins in water-containing naphtha streams.

## 2. Materials and Methods

### 2.1. Materials

An industrial naphtha provided by Nexen (A CNOOC Limited Company) in Calgary, AB, Canada, was used as feedstock. This feedstock was hydrogenated in a pilot plant to reduce olefin content. Virgin Athabasca Bitumen was supplied by Japan Canada Oil Sands Limited Company (JACOS), Calgary, AB, Canada. The physical properties of the Athabasca bitumen provided by JACOS are shown in Table 1. The sample is a typical highly viscous Alberta bitumen having an API gravity of 9.5. The bitumen was originally produced from the steam-assisted gravity drainage (SAGD) process and was further dehydrated to reach a final water content of 0.4 wt.%.

### 2.2. Preparation of Precursor-10 wt.% Mo_2_C/γ-Al_2_O_3_ Catalytic Spheres Using the Sucrose Route

In a 2 L glass beaker, 162 g of ammonium heptamolybdate tetrahydrated (NH_4_)_6_Mo_7_O_24_·4H_2_O (AHM) analytical reagent (81–83% MoO_3_ basis, CAS:12054-85-2) from Sigma-Aldrich, Oakville ON, Canada was dissolved in 1100 g of deionized water with magnetic stirring at 300 rpm. After complete dissolution, 92 g of household sucrose (C_12_H_22_O_11_) was added to the AHM solution under magnetic stirring. The mixture was homogenized for 15 min at room temperature, followed by placing the solution in an oven at 100 °C for 40 h until a dark green, paint-like solution was obtained [32]. This step aimed to prepare the wet Mo_2_C precursor with a C/Mo ratio of 3.5 for subsequent impregnation. Subsequently, 70.5 g of SASOL alumina spheres (3.0 mm diameter, product code 610110, Lot number TKA363, Calgary, AB, Canada) were impregnated with a solution created by diluting 20 g of the wet Mo_2_C precursor in 20 g of deionized water. The black spheres were dried at 150 °C for 18 h. The resulting 85.8 g of supported dried precursor 10 wt.% Mo_2_C/γ-Al_2_O_3_ was placed in a sealed plastic container for storage and future use. To produce the active cubic Mo_2_C phase, the spheres were thermally treated at 500 °C for 12 h in a fixed bed reactor under H_2_ atmosphere at a flow rate of 120 sccm [32].

### 2.3. Pilot Plant Catalytic Tests

**Figure 1:**
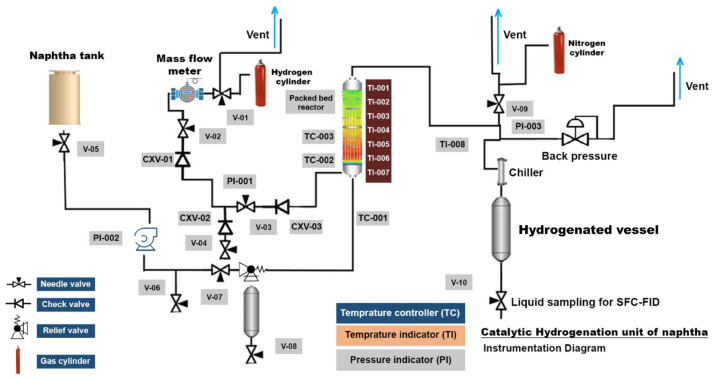
Piping and instrumentation diagram of the pilot plant hydrogenation unit used for olefin hydrogenation tests on industrial naphtha feedstock.

Reactivity experiments were conducted in a bench-scale pilot plant featuring an up-flow open tubular reactor [37,38], as illustrated in Figure 1. To prevent gum or deposit formation in the entrance lines, the naphtha and H_2_ feed lines were maintained at room temperature. The reactor temperature was varied at three levels (200, 250, and 280 °C) with a fixed H_2_ flow rate of 33 sccm. Naphtha flow rate control was achieved through an Isco pump, while a stainless steel up-flow reactor (1/2-inch internal diameter) was loaded with 10 g of 10 wt.% Mo_2_C/γ-Al_2_O_3_ catalyst. A thermocouple placed in the reactor’s packed bed wall, along with an Omega internal 7-point profile thermocouple, recorded temperature profiles. The latter had five points within the catalyst bed, and the other two were positioned in the middle of the upper and bottom packing material bed (SiC). The exit line of the reactor, set at room temperature, facilitated product stream condensation. Reactor pressure was regulated using a Swagelock back-pressure valve, set at 3.44 × 10^6^ Pa. Maintaining a desired weight hourly space velocity (WHSV = 0.43 h^−1^), the naphtha flow rate was controlled. WHSV was universally defined under pumping conditions, as expressed in Equation (1).

(1) $$WHSV\;[h^{-1}]=\frac{Naphtha\;mass\;flow\;rate\;(g/h)}{Catalyst\;mass\;(g)}$$

The outlet gas stream flow rate was measured with a wet gas flow meter, and the gas collected in a sampling bag, which underwent analysis to ensure no hydrocarbons were entrained within the H_2_ effluent. The reaction product stream, after passing through a chiller, was collected for olefin quantification using supercritical fluid chromatography coupled with a flame ionization detector (SFC-FID) analysis. Liquid samples were analyzed online every 4 h using SFC-FID. The experiment that produced the least olefin content was then selected for another reactivity test in the presence of both H_2_ and steam to test the stability of catalyst under steam environment and to simulate the hydrogenation of naphtha produced from the hot separator in a CSC process. It is worth mentioning that in all experiments, the catalyst had to be activated prior to the naphtha hydrogenation tests. Then, 10 g of the precursor—10 wt.% Mo_2_C/γ-Al_2_O_3_ spheres were hydrothermally treated under atmospheric H_2_ flow of 120 sccm at 500 °C for 12 h.

### 2.4. Characterization Methods

Scanning electron microscopy coupled with energy dispersive X-ray (SEM-EDX, FEI, Calgary, AB, Canada) mapping was conducted on the 10 wt.% Mo_2_C/γ-Al_2_O_3_ catalyst. A minute quantity of the catalyst samples was affixed to an adhesive double-sided carbon tape secured on the metallic sample holder, which was subsequently tapped to eliminate excess powder. SEM images were captured using a field emission Quanta 250 electron microscope (FEI) with a voltage of 20 kV and a spot size of 3.0. Elemental identification on the material’s surface was achieved by employing an EDX detector.

For the determination of olefin content pre- and post-naphtha hydrogenation, supercritical fluid chromatography coupled with a flame ionization detector (SFC-FID; Selerity Technologies Inc., Salt Lake City, UT, USA) was employed. The SFC equipment, supplied by Selerity Technologies (Selerity Technologies Inc., Series 4000 SFC, Salt Lake City, UT, USA), utilized helium as the carrier gas. Initially, the ASTM D-5186 method [39] assessed the wt.% aromatics and non-aromatics in the feedstock by passing the sample through a packed silica column, as depicted in Figure S1 in the Supplementary Material section. ASTM D-6550 method [40] was also applied to separate saturates, aromatics, and olefins, utilizing silica and a 10% silver/silica packed column. A 1 mL liquid sample was collected, withdrawn by an auto-sampler, and injected into the SFC apparatus through a 60 nL Valco valve model. Quantification of saturates, aromatics, and olefins (SAO analysis) was executed using a flame ionization detector (FID), and data were collected and processed with EZChrom Elite data acquisition software, as shown in Figure S2 in the Supplementary Material.

Additionally, gas chromatography coupled with mass spectroscopy and a flame ionization detector (GC-MS-FID) was employed for paraffins, olefins, naphthenes, and aromatics (PONA) analysis of the feedstock. HP6890 series GC system (Hewlett Packard) with an integrated PONA column (length: 50 m, inner diameter: 200 µm, film thickness: 0.5 µm) and a flame ionization detector (FID) was utilized, following the ASTM D-5134 method [41], as shown in Table S1.

The metal contents of both JACOS Alberta bitumen and Nexen Naphtha were quantified using inductively coupled plasma (ICP) spectroscopy [34]. The samples were placed into appropriate Teflon vessels, and a mixture of hydrochloric and nitric acids was introduced. Subsequently, the vessels were sealed and subjected to heating in a MARS 6 microwave digestion apparatus manufactured by CEM for a complete cycle to extract and dissolve the metals present in the solids into the acid solution. The resulting solutions underwent ICP analysis using an Iris Intrepid II XDL spectrometer manufactured by Thermo Electron Corporation (Waltham, MA, USA) [34].

## 3. Results and Discussion

### 3.1. Characterization Results

Table 2 presents the physical properties and chemical composition of the investigated naphtha. It is evident that naphtha contains nearly 1 wt.% sulfur, a notably high value for naphthas. Group-type analysis, conducted for saturates, aromatics, and olefin content (wt.%) using SFC-FID, aligns closely with the findings derived from GC-MS-FID. Additionally, the concentration of heavy metals determined via inductively coupled plasma (ICP) in the naphtha was nearly negligible.

**Figure 2:**
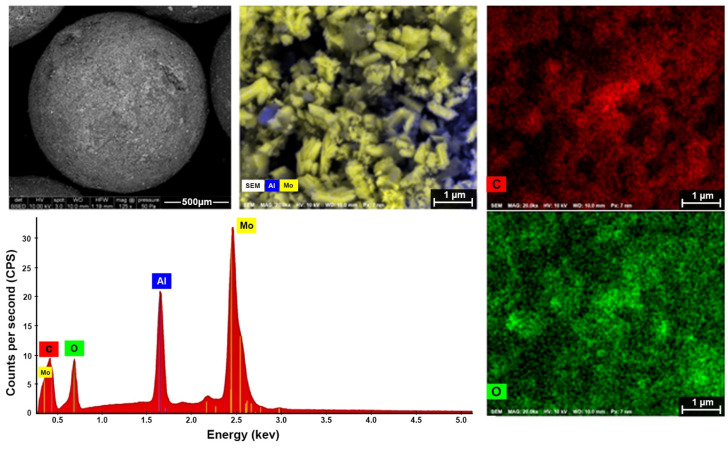
Scanning electron microscope–energy dispersive X-ray (SEM-EDX) mapping images for 10 wt.% Mo_2_C/γ-Al_2_O_3_ spheres.

Furthermore, Figure 2 illustrates SEM images and EDX mapping for the 10 wt.% Mo_2_C/γ-Al_2_O_3_ spheres. The Mo_2_C phase exhibits uniform distribution across the Al_2_O_3_ spheres, with all constituent elements (Mo, C, Al, and O) observed on the catalyst surface. The carbide structure was also confirmed by X-ray diffraction, as depicted in Figure S3.

### 3.2. Catalytic Hydrogenation Tests of Naphtha on 10 wt.% Mo_2_C/γ-Al_2_O_3_ Spheres

**Figure 3:**
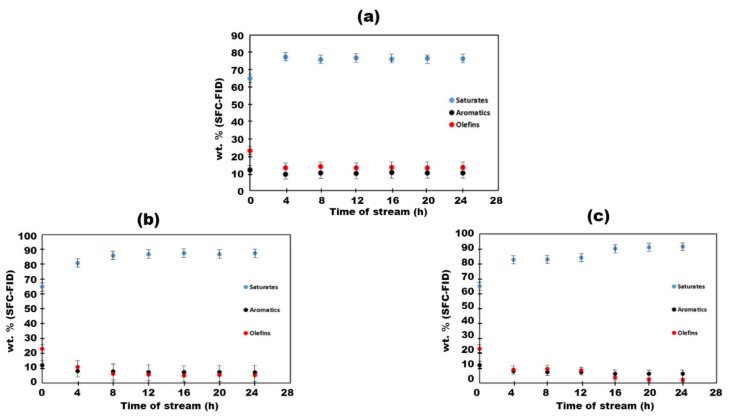
Saturates, aromatics, and olefins time–supercritical fluid chromatography (SFC) profiles during the hydrogenation of naphtha over 10 wt.% Mo_2_C/γ-Al_2_O_3_ spheres at 3.44 × 10^6^ Pa, 33 sccm H_2_ flow and WHSV = 0.43 h^−1^: (**a**) 200 °C, (**b**) 250 °C, and (**c**) 280 °C.

**Figure 4:**
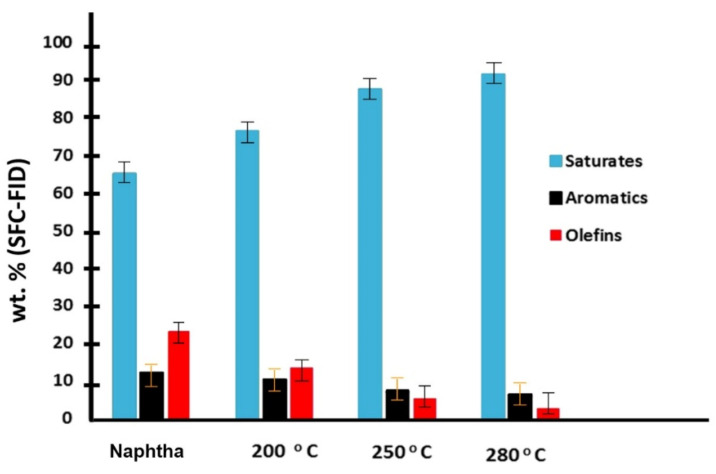
Bar chart at the tested temperatures for the final saturates, aromatics, and olefin content (wt.%) in naphtha after hydrogenation over 10 wt.% Mo_2_C/γ-Al_2_O_3_ spheres for 24 h (pressure = 3.44 × 10^6^ Pa, WHSV = 0.43 h^−1^, H_2_ flow = 33 sccm).

The naphtha hydrogenation experiments conducted with 10 wt.% Mo_2_C/γ-Al_2_O_3_ catalyst demonstrated a notable efficacy in the removal of olefins. The alterations in saturates, aromatics, and olefin content (wt.%) after naphtha hydrogenation at 200 °C, 250 °C, and 280 °C are depicted in Figure 3. At 200 °C and after 4 h, a decrease in aromatics content from 12% to 10% was observed in Figure 3a, concomitant with a reduction in olefin content from 23% to 13%. Saturates, as anticipated, exhibited an increase from 65% to 76% because of the conversion of olefins to their saturate counterparts. Elevating the temperature to 250 °C resulted in a notable decline in aromatic content from 12% to 7%, affirming the activity of the 10 wt.% Mo_2_C/γ-Al_2_O_3_ spheres in aromatic hydrogenation at higher temperatures. Additionally, the quantity of olefins decreased from 23% to 5%, as indicated in Figure 3b. At the highest temperature (280 °C), the aromatic content exhibited no significant decrease, with a final content of approximately 6%, without signs of catalyst deactivation, as depicted in Figure 3c. A summary of all hydrogenations results over 10 wt.% Mo_2_C/γ-Al_2_O_3_ spheres is presented in Figure 4. Olefin content was reduced to 2.5% at 280 °C, indicating that incorporating this hydrogenated naphtha as a diluent in a ratio of 30 wt.% to bitumen would result in a total final olefin content of 0.75 wt.% (i.e., <1 wt.%), thereby meeting pipeline specifications for oil transportation. This aspect will be further discussed in the preceding section.

### 3.3. Incorporating Hydrogenated Naphtha as a Diluent into JACOS Athabasca Bitumen to Meet Pipeline Specifications

**Figure 5:**
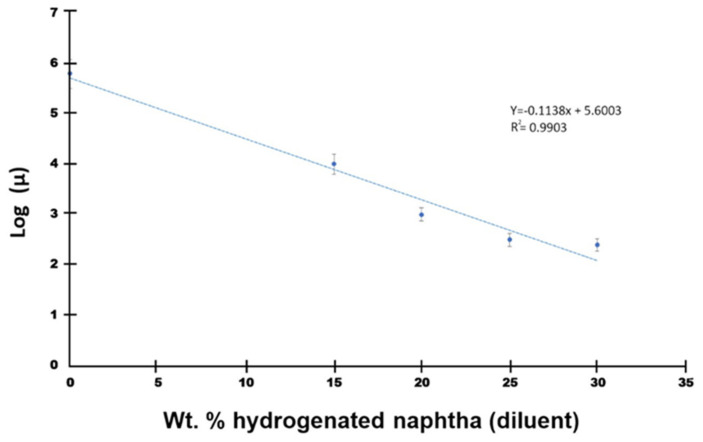
Linear plot for log (µ) at 7.5 ^°^C versus wt.% diluent added to JACOS bitumen.

The naphtha product obtained from catalytic hydrogenation over 10 wt.% Mo_2_C/γ-Al_2_O_3_ spheres at 280 °C and 500 psig for 24 h was introduced into JACOS Athabasca bitumen to decrease its viscosity for pipeline conveyance. Figure 5 illustrates the linear regression analysis depicting the logarithmic relationship between viscosity (µ) and the wt.% of diluent (i.e., hydrogenated naphtha) added to the bitumen. At 7.5 °C, the final viscosity of the blend (i.e., dilbit) with 30 wt.% diluent was approximately 220 cP (i.e., <320 cP), meeting the specified criteria for dilbit transportation for Canadian pipelines. Moreover, the ultimate olefin content was <1 wt.%, as elaborated earlier in Section 3.2. The final *p*-value of the dilbit exceeded 1.8, and the API gravity value measured was 21.9.

### 3.4. Catalytic Hydrogenation Tests of Naphtha on 10 wt.% Mo_2_C/γ-Al_2_O_3_ Spheres in Presence of Steam Simulating the Hot Separator Stream

**Figure 6:**
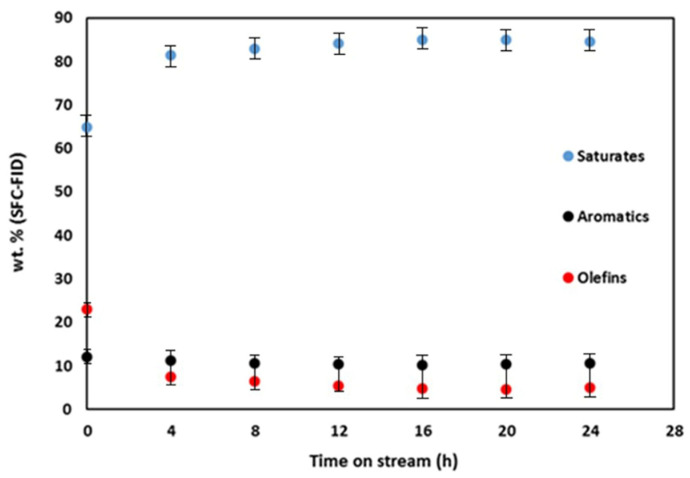
Saturates, aromatics, and olefins time–supercritical fluid chromatography (SFC) profiles during the hydrogenation of naphtha in the presence of steam over 10 wt.% Mo_2_C/γ-Al_2_O_3_ spheres at 280 °C, 3.44 × 10^6^ Pa, 33 sccm H_2_ flow and WHSV = 0.43 h^−1^. (Water/naphtha mass flow rate ratio = 5:3).

In the preceding sections, we evaluated the catalyst activity for olefin hydrogenation in naphtha, identifying 10 wt.% Mo_2_C/γ-Al_2_O_3_ spheres at 280 °C as yielding naphtha with minimal olefin content. Consequently, the same catalyst was selected for olefin hydrogenation under identical conditions (i.e., 280 °C and 3.44 × 10^6^ Pa) but with the introduction of steam. This facilitated the assessment of catalyst stability in a steam environment and the simulation of naphtha stream hydrogenation containing water, as encountered in the hot separator following the CSC process. Drawing from prior pilot plant investigations, a water/naphtha mass flow rate ratio of 5:3, reflective of typical CSC unit naphtha–water mixture compositions, was chosen. Despite the presence of steam in the reaction, olefin hydrogenation proceeded, resulting in a final olefin content reduction from 23% to 5%, with a concurrent aromatics content reaching 10%. The findings presented in Figure 6 unequivocally demonstrate the catalyst’s resilience to both steam and sulfur, endorsing its viability for commercialization as a selective hydrogenating catalyst for olefins in light petroleum distillates (e.g., naphthas and gasolines) under moderate temperatures, pressures, and steam conditions.

## 4. Conclusions

It has been demonstrated that our internally developed catalyst, synthesized via the sucrose route, effectively reduced the olefin content in an industrial naphtha feedstock. Spheres composed of 10 wt.% Mo_2_C/γ-Al_2_O_3_ exhibited notable catalytic activity and stability during olefin hydrogenation. The supported carbide catalyst achieved a final olefin content reduction to 2.5 wt.%, enabling compliance with pipeline specifications (i.e., <1 wt.% olefins) for oil pumping by incorporating the resulting hydrogenated naphtha as a diluent in Athabasca bitumen at a 30 wt.% ratio. Furthermore, the catalyst exhibited robust tolerance to sulfur present in the naphtha for a duration of 96 h, manifesting no indications of catalyst deactivation. Lastly, the supported molybdenum carbide demonstrated exceptional stability and high activity in steam environments, suggesting its potential utility for hydrogenating naphtha streams containing water derived from the hot separator post-CSC process.

## Figures and Tables

**Table 1:** JACOS Athabasca Bitumen chemical and physical properties.

Analysis Bitumen		JACOS Athabasca
API gravity		9.5
Initial boiling point (°C) by simulated distillation *^a^*		171
wt.% residue > 545 °C by simulated distillation *^a^*		50
viscosity at 7.5 °C [cP]		444,324
water content [wt.%]		0.4
carbon [wt.%]		83.5
hydrogen [wt.%]		10.6
sulfur [wt.%]		4.8
nitrogen [wt.%]		0.4
Mo [ppm]		12.1
Ni [ppm]		78.7
V [ppm]		191
W [ppm]		1
Fe [ppm]		10
SARA *^b^*	saturates [wt.%]	15
	aromatics [wt.%]	48
	resins [wt.%]	25
	asphaltenes-C_7_ [wt.%]	12
Total acid number (TAN) *^c^*		2.6

*^a^* Determined using ASTM D-7169 method [34]. *^b^* Determined as in Carbognani et al. [35]. *^c^* Determined using ASTM-D664 method [36].

**Table 2:** Physical and chemical composition of naphtha feedstock.

Nexen’s Cracked Naphtha	Physical Properties and Chemical Composition
Density at 20 °C (g/cm^3^)	0.784
Initial boiling point (°C) by SIMDIST	−11 °C
Final boiling point (°C) by SIMDIST	270 °C
Sulfur content (ppm)	10,700 (~1 wt.%)
Metal analysis (ICP)	4.5 ppm Fe
SFC-FID analysis (wt.%)/ASTM D-6550 [40]	65% saturates, 12% aromatics, 23% olefins
SFC-FID analysis (wt.%)/ASTM D-5186 [39]	90% non-aromatics, 10% aromatics
GC-FID-MS analysis (wt.%) [41]	66% saturates, 11% aromatics, 23% olefins

## Data Availability

Data are contained within the article.

## Supplementary Materials

The following supporting information can be downloaded at: https://www.mdpi.com/article/10.3390/ma17102278/s1, Figure S1. Normalized supercritical fluid chromatography (SFC) chromatogram for Nexen naphtha feedstock using ASTM D-5186 method. Figure S2. Normalized supercritical fluid chromatography (SFC) chromatogram for Nexen naphtha feedstock using ASTM D-6550 method. Table S1. Olefinic molecules present in Nexen naphtha, as determined by GC-MS-FID (ASTM D-5134). Figure S3. X-ray diffraction (XRD) diffractogram of the activated Mo_2_C prepared by the sucrose route.
