# Supplementary material for: Enhanced Catalytic Hydrogenation of Olefins in Sulfur-Rich Naphtha Using Molybdenum Carbide Supported on γ-Al2O3 Spheres under Steam Conditions: Simulating the Hot Separator Stream Process
Source: Materials (Basel). 2024 May 11;17(10):2278. doi: 10.3390/ma17102278 (PMC11123070; doi:10.3390/ma17102278)
Supplement: Supplementary file 1 [file materials-17-02278-s001.zip › materials-2951636-supplementary.pdf]

Appendix A. Supplementary data

Enhanced Catalytic Hydrogenation of Olefins in Sulfur-rich Naphtha Using Molybdenum Carbide Supported on γ-Al_2O_3 Spheres under Steam Conditions: Simulating the Hot Separator Stream Process

Hadj Abbas Abbas[1], Zahra Asgar Pour[2*], Mohammed S. Alnafisah[3], Pablo Gonzalez Cortes[4,5], Mustapha El Hariri El Nokab[6], Ahmed Elshewy[7], Khaled O. Sebakhy[8,9*]

1 Laboratoire de géologie du Sahara, Université Kasdi Merbah Ouargla, Ouargla, Algeria.

2* Research and Development Department, Kisuma chemicals, Billitonweg 7, 9641 KZ Veendam, The Netherlands.

3 King Abdulaziz City for Science and Technology (KACST), Riyadh 12354, Saudi Arabia.

4 Engineering and Technology Institute Groningen (ENTEG), University of Groningen, Nijenborgh 4, 9747 AG Groningen, The Netherlands.

5 Laboratorio de Nanocelulosa y Biomateriales, Departamento de Ingeniería Química, Biotecnología y Materiales, Facultad de Ciencias Físicas y Matemáticas, Universidad de Chile, Avenida Beauchef 851, Santiago, Chile 8330111.

6 Department of Chemistry, Michigan State University, East Lansing, MI 48824, USA.

7 Department of Pharmaceutical Organic Chemistry, Faculty of Pharmacy, Cairo University, 11562 Cairo, Egypt.

8* Laboratory for Chemical Technology (LCT), Department of Materials, Textiles and Chemical Engineering, Ghent University, Technologiepark 125, 9052 Ghent, Belgium.

9*Department of Chemical and Petroleum Engineering, University of Calgary, Calgary, Alberta, Canada T2N 1N4.

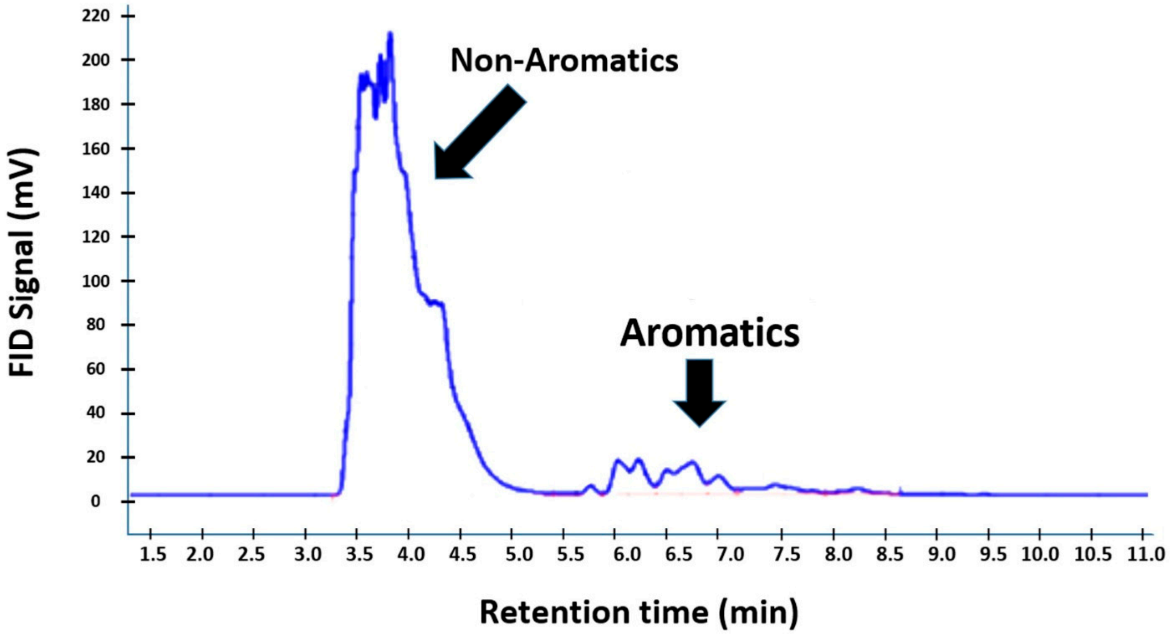

Figure S1. Normalized SFC chromatogram for Nexen naphtha feedstock using ASTM D-5186 method.

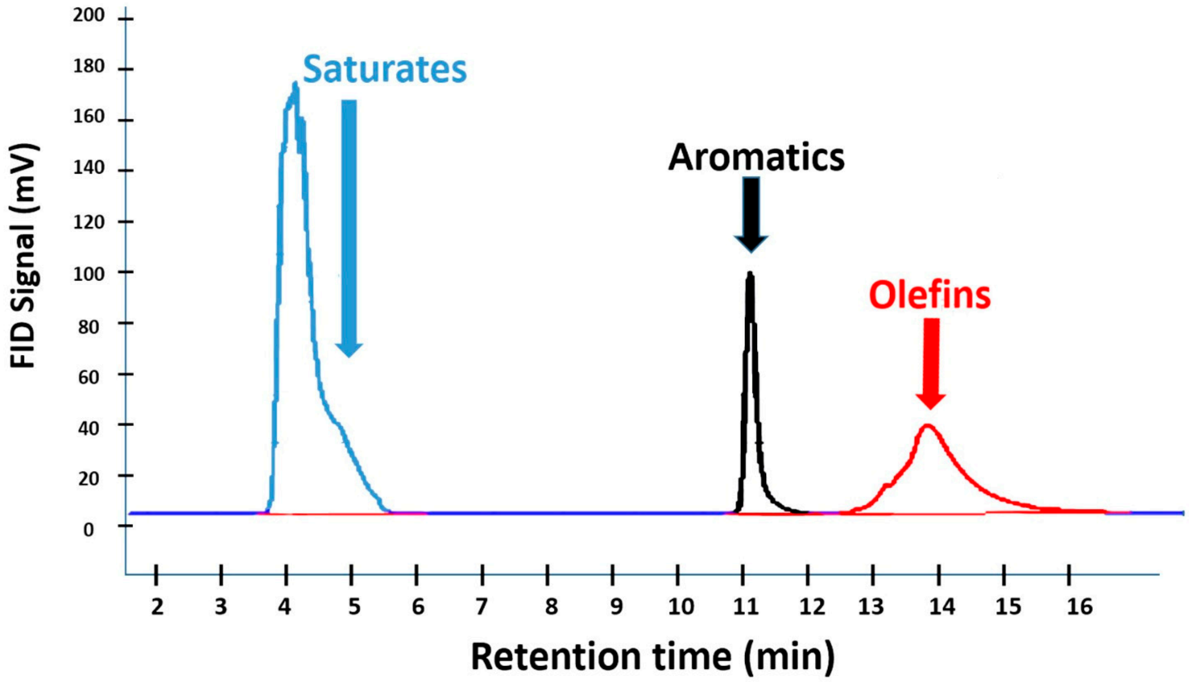

Figure S2. Normalized SFC chromatogram for Nexen naphtha feedstock using ASTM D-6550 method.

Appendix A. Supplementary data

Table S1. Olefinic molecules present in Nexen naphtha, as determined by GC-MS-FID (ASTM D-5134).

2-methyl, 1-propene	2-hexene (Z)	2-heptene (Z)
1-butene (Z)	3-methyl, 2-pentene (E)	1,2,3-trimethyl cyclopentene
2-butene (Z)	2,4-dimethyl-1-pentene	2-ethyl cyclopentene
2-methyl, 1-butene	1-methyl cyclopentene	4-methyl-3-heptene
1-pentene	3-methyl, 1-hexene	1,2,3-trimethyl cyclopentene
2-methyl, 1-butene	2,4-dimethyl-2-pentene	5-methyl-3-heptene
2-pentene (E)	2-methyl-3-hexene	1-methyl cyclohexene
2-pentene (Z)	4-methyl-1-hexene	6-methyl-2-heptene
2-methyl, 2-butene	4-methyl-2-hexene	6-methyl-2-heptene
Cyclopentene	Cyclohexene	2,5-dimethyl -2,4-hexadiene
4-methyl, 1-pentene	4,4-dimethyl-2-pentene	1-Octene
3-methyl, 1-pentene	1-heptene	1,2,3-trimethyl cyclopentene
2,3 dimethyl-butene	3-methyl-3-hexene	4-Octene (E)
2-methyl, 1-pentene	4,4-dimethyl cyclopentene	Methyl-ethyl cyclopentene
1-hexene	3-heptene	3-Octene (E)
3-hexene (E)	2-methyl, 2-hexene	2-Octene (E)
3-hexene (Z)	3-methyl, 3-hexene (E)	1,2,3 trimethyl cyclopentene
2-hexene (E)	2-heptene	2-Octene (Z)
4-methyl, 2-pentene	3,5-dimethyl cyclopentene	2,6-dimethyl-3-heptene
3-methyl, cyclopentene	3-methyl-2-hexene (Z)	1-methyl-thio-1-butene
3-methyl, 2-pentene		2-ethyl cyclohexene
3-methyl cyclopentene		1-ethyl cyclohexene
		2,6-dimethyl-2-heptene
		1-Nonene
		2-Nonene
		3-Nonene
		5-Nonene
		4-Nonene
		4-Decene
		1-methyl-2-decene
		2-methyl-3-decene

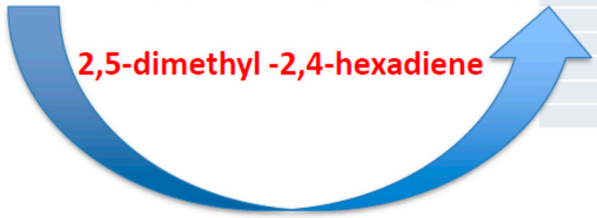

36

References

(1) Badoni, R. P.; Bhagat, S. D.; Joshi, G. C. Analysis of olefinic hydrocarbons in cracked petroleum stocks: a review. *Fuel* **1992**, *71*(5), 483-491. https://doi.org/10.1016/0016-2361(92)90144-D.

Appendix A. Supplementary data

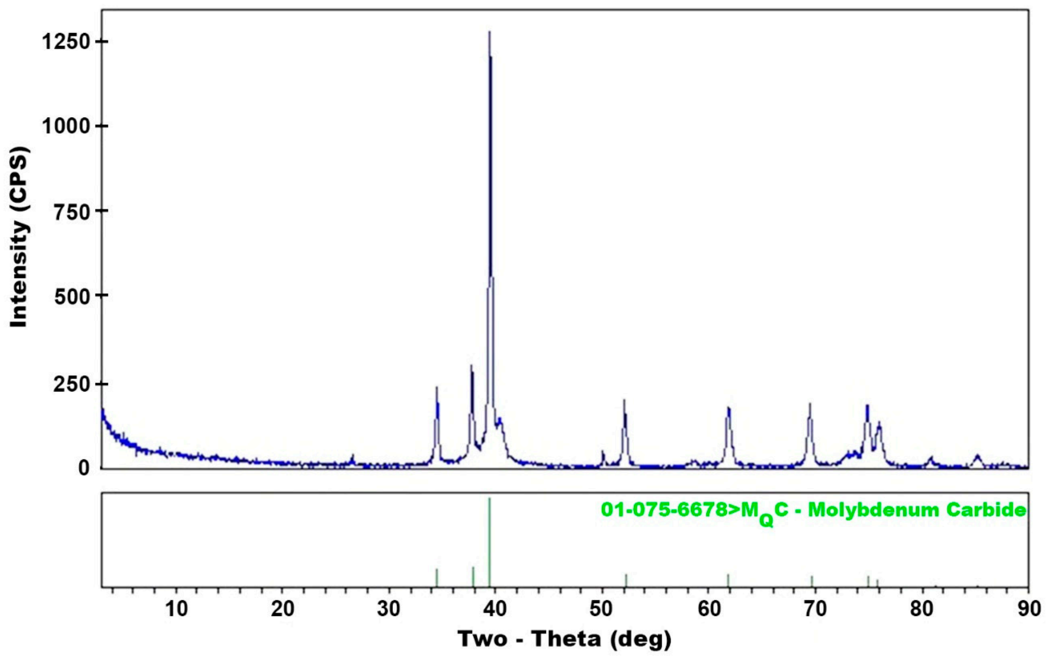

Figure S3. XRD diffractogram for the activated Mo_2C prepared by the sucrose route.
